# Fault State Identification Method with Noise Robustness of Dry Gas Seals Based on Sample-Augmented MA1D-ResNet

**DOI:** 10.3390/s25227005

**Published:** 2025-11-16

**Authors:** Jinlin Chen, Jiahao Li, Xuexing Ding, Wei Xu, Pengju Li, Zhihao Xia

**Affiliations:** School of Petrochemical Technology, Lanzhou University of Technology, Lanzhou 730050, China; jinlinchen119@163.com (J.C.); 232085702005@lut.edu.cn (J.L.); 20050032@lut.edu.cn (X.D.); 24208370003@lut.edu.cn (P.L.); 242085702001@lut.edu.cn (Z.X.)

**Keywords:** dry gas seal, acoustic emission, multi-scale attention, residual network, sample augmentation, fault diagnosis

## Abstract

Dry gas seals are widely used in the petrochemical industry for shaft end sealing of compressors, pumps and other equipment involving flammable, explosive, toxic and harmful media. To address the challenge of accurately identifying the fault states of dry gas seals under strong noise interference, this paper proposes a Multi-scale Attention 1D Residual Network (MA1D-ResNet) model based on sample augmentation. First, a dry gas seal acoustic emission (AE) test rig was built to collect non-stationary AE signals. The training dataset was expanded to five times its original size through data segmentation and Gaussian noise injection, significantly enhancing the model’s generalization capability in the data-input domain and training process. Then, the proposed model incorporates a Multi-scale Dual Attention Module (MDAM) into the ResNet18 architecture: it employs 1D convolutions to process temporal signals directly, avoiding feature loss, and integrates MDAM after the first convolutional layer and the Stage1 layer to strengthen fault feature extraction. Finally, experimental results demonstrate that the proposed model achieves an average accuracy of 99.8571% in classifying seven fault states (significantly outperforming five comparative models including CNN, ResNet, and ResNet-CBAM), with 100% recognition rate for five of the fault categories. The proposed model exhibits outstanding noise robustness, maintaining an accuracy of 92.43% under strong noise conditions of −6 dB. This study provides a highly robust solution for the intelligent fault diagnosis of dry gas seals in complex noise environments.

## 1. Introduction

Dry gas seals are widely used in equipment such as compressors, pumps, and stirred reactors due to their advantages of minimal wear during operation, low energy consumption, small leakage rates, and long service life [1]. However, it is challenging to completely prevent failures or abnormal conditions in dry gas seals during operation. For instance, the intrusion of liquids or contaminants into the sealing faces, contamination of the gas supply, metal fatigue induced by long-term operation, and dry friction during start-stop cycles can all lead to failures such as wear of the sealing faces. Traditional fault diagnosis methods for this equipment involve indirectly assessing the operational status by monitoring parameters such as the leakage rate, temperature rise, or pressure of the sealing device during operation. The interpretation of these parameters is heavily reliant on expert experience and domain knowledge. Moreover, these methods suffer from low automation levels, fail to directly reflect the equipment’s operational health, and are inadequate for achieving predictive maintenance.

Acoustic emission (AE) technology, which captures acoustic wave signals generated by energy release events such as the formation and propagation of internal microcracks, friction, and leakage in materials, enables early identification and real-time monitoring of potential structural faults. It has become one of the core technologies in fault diagnosis and is widely applied in the monitoring and diagnosis of mechanical equipment, including wind turbines [2,3], gearboxes [4,5], milling machine [6], and rolling bearings [7,8].

In research on fault diagnosis of dry gas seals, Williams et al. [9] were among the first to apply AE measurements to monitor the sliding contact behavior in mechanical end face seals. They conducted tests under various operating conditions, including normal and dry sliding contacts, as well as conditions with high leakage rates and elevated temperatures, to assess the stability of the sliding contact. Towsyfyan et al. [10] developed a mathematical model for the AE energy release of mechanical seals across different tribological states. The model enables condition monitoring and fault diagnosis based on AE signal characteristics. Manuel et al. [11] used AE to detect and analyze fault-inducing friction mechanisms within a mechanical seal. The research team led by Huang et al. [12,13,14,15] has conducted extensive work on dry gas seal fault diagnosis using AE technology. In their early studies, they used AE sensors to capture signals during the startup process and successfully distinguished friction signals at the sealing faces through power spectrum analysis and band-pass filtering. Ding et al. [16,17] set up experimental test rigs and AE measurement systems to monitor AE signals under various typical fault conditions. They employed machine learning algorithms to differentiate fault types, effectively isolating and identifying fault sources, while enriching the dry gas seal fault database. Acoustic emission technology has shown a rather significant role and great application potential in fault diagnosis.

The field of intelligent fault diagnosis generally follows two paradigms: model-driven and data-driven approaches. Model-driven methods (mechanism-based modeling and state inference) are interpretable and verifiable when physical laws are clear and parameters are identifiable [18,19]. However, dry gas seals involve friction–fluid–structure coupling and operate under time-varying conditions, constructing precise models of such complex dynamical systems is often impractical, thereby restricting their widespread application. In contrast, data-driven methods learn directly from operational data, making them highly suitable for scenarios where system models are unknown or difficult to establish, and they have become the mainstream approach [20,21,22,23,24,25]. For instance, Pacheco et al. [20] and He et al. [22] proposed 1D-CNN-based deep transfer learning frameworks to address challenges like cross-domain adaptation and few-shot learning in rotating machinery fault diagnosis. To enhance the robustness of traditional methods (e.g., manual feature extraction with SVM) under noise and varying operational conditions, extensive research has been conducted. Peng et al. [26] introduced a deep 1D residual convolutional network (Der-1DCNN), which employs residual learning and wide kernels to achieve end-to-end, high-accuracy diagnosis. Similarly, Zhang et al. [27] developed a multi-level information fusion method based on 1D-CNN that adaptively combines vibration and acoustic signals for improved fault recognition. Despite these successes, further research has uncovered inherent limitations in CNNs. For example, the convolutional kernel has a limited local receptive field, requiring many convolutional layers to capture global information [28].

To address these issues, ResNet was introduced. It utilizes residual connections to mitigate the vanishing gradient problem, enabling the construction of deeper and more powerful models. ResNet has been widely applied in fault diagnosis for centrifugal pumps [29], batteries [30], bearings [31], and sensors [32]. However, conventional deep learning models often struggle to focus on critical features when processing non-stationary signals with low signal-to-noise ratios, rendering them susceptible to noise interference. To enhance feature selectivity, researchers have integrated attention mechanisms with neural networks. For instance, hybrid CNN–RNN models have been developed to capture spatiotemporal dependencies [33]; the SE channel attention module has been incorporated to adaptively weight informative channels [34]; and compact architectures such as PDSResNet-AM have been proposed, which combine parallel depthwise separable convolution with CBAM to maintain efficiency under variable loads, noise, and speed fluctuations [35]. Building on this line, noise-robust and multi-scale designs have further improved performance. Han et al. [36] proposed a multi-scale attention network, and Chen et al. [37] introduced an ECA-enhanced soft-thresholding strategy to strengthen noise-resilient feature learning. Nevertheless, fixed-size convolutional kernels may still be sub-optimal for faults spanning disparate temporal scales. Moreover, intelligent fault diagnosis for dry gas seals remains relatively unexplored, primarily due to challenges such as ambiguous failure modes, intense background noise, and the absence of standardized datasets.

To address these challenges, this study designed and constructed a non-contact rotational acoustic emission test rig to collect non-stationary AE signals from dry gas seals under various fault conditions. We propose a novel deep learning model, MA1D-ResNet, based on a 1D-ResNet architecture, which extracts features directly from the raw one-dimensional AE signals. To further improve model performance, Gaussian noise injection was used for data augmentation. This approach expands the training dataset by simulating sensor interference encountered in real operational environments and enhances the model’s robustness and generalization in noisy conditions.

The main contributions of this work are as follows:

1. Experimental Design and Data Enhancement: We designed seven common operational conditions, including four structural fault conditions of the seal face and three operational states during startup: dry friction (50 rpm), mixed lubrication (600 rpm), and stable operation (1000 rpm). AE technology was used to monitor the seal status. A data augmentation strategy involving noise injection was implemented to expand the sample set and enhance the noise robustness of the input data.
2. Novel Diagnostic Model: We propose the MA1D-ResNet model for dry gas seal fault diagnosis. The model incorporates a multi-scale spatial attention mechanism, integrating three different convolutional kernels for signal feature identification, combined with a channel attention module and ResNet architecture. This design enables accurate extraction of multi-scale fault-related features from AE signals.
3. Comprehensive Validation: We validated the data augmentation method and the MA1D-ResNet model using a self-constructed dataset. Comparative experiments with five existing models under identical parameter settings, including accuracy, loss, and noise immunity tests, demonstrate that our method offers superior diagnostic performance and better robustness to noise.

The rest of this article is organized as follows: Section 2 details the proposed MA1D-ResNet model. Section 3 presents the experimental results. Section 4 summarizes the study.

## 2. Proposed Method for Fault Diagnosis of Dry Gas Seal

### 2.1. One-Dimensional Residual Neural Network

The Residual Neural Network (ResNet) is a deep learning architecture first proposed by He et al. [38] in 2015. It addresses the issues of vanishing and exploding gradients in deep neural networks by employing residual blocks, which enables the training of much deeper networks.

**Figure 1:**
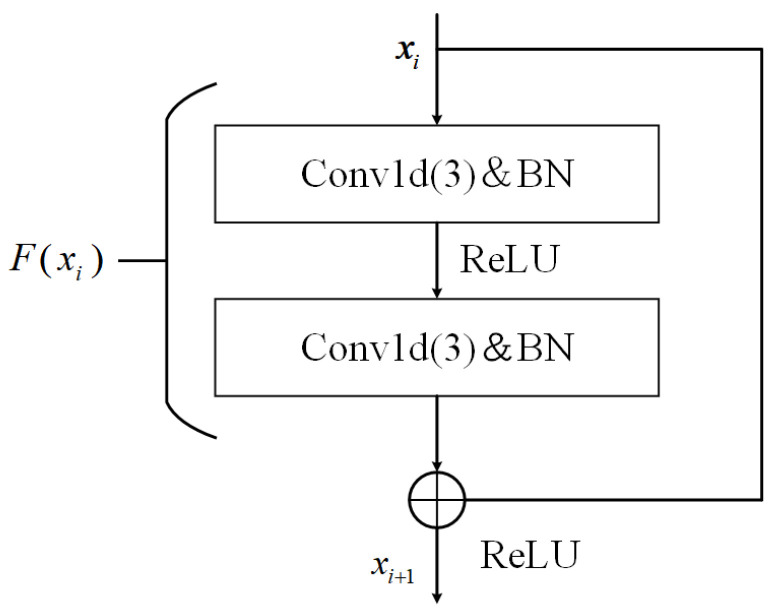
The structure of BasicBlock.

In this study, the sample data consists of one-dimensional time-series signals, directly acquired from the data acquisition system. Therefore, one-dimensional convolution with a kernel size of 3 is used to capture the features of these signals. Each block contains several sequential operations, including convolutional layers, batch normalization, and ReLU activation functions. Importantly, identity skip connections are integrated with these operations. The formula for the basic block is as follows:

(1) $$x_{i+1}=f\;\left(F\left({h(x}_{i}),{\;W}_{i}\right)\;+\;x_{i}\right)$$

where *h*(*x_i_*) is the shortcut connection; the function *F*(∙) is the residual block mapping, which represents the learned residual; *W_i_* is the network parameter; *f*(∙) is the ReLU activation function. The fundamental building block of the ResNet residual network used in this paper is the BasicBlock. A BasicBlock consists of two convolutional layers, and its structure is illustrated in Figure 1.

### 2.2. Attention Module

#### 2.2.1. Channel Attention Module

**Figure 2:**
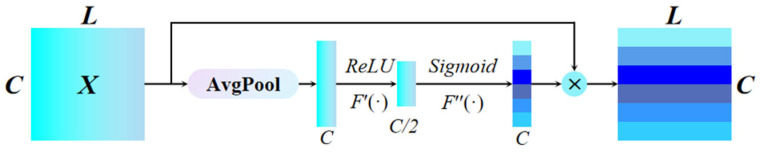
Channel Attention Module.

The Channel Attention Module (CAM) is designed to model the interdependencies between channels, enhancing the network’s sensitivity to different features. As a result, it adaptively recalibrates the significance of feature representations across channels, improving the model’s discriminative power. The basic structure of the CAM is shown in Figure 2.

Assuming the input to the CAM is $X\;=\;[x_{1},x_{2},x_{3},\;\ldots \;x_{C}]$, Where X represents the combination of the input sample channels $\left(x_{i}{\in \;R}^{\;L\times 1}\right)$. First, CAM applies global average pooling to compress the global temporal information, and generates channel-wise statistics vector $y\;(y\in \;R^{1\times C})$. The *i*-th element of *y* is calculated by:

(2) $${y\;}_{i}\;=\;\mathrm{Avgpool}\left(x_{i}\right)\;=\;\frac{1}{1\;\times \;L}\sum_{j=1}^{L}x_{i}\left(j\right)$$

The compressed global temporal information is embedded into the vector *y*. This pooled output is then passed through a convolutional layer to generate a channel calibration vector, denoted as *y′*, which is defined by the following formula:

(3) $$y^{\prime }\;=\;\sigma \;(F^{\prime \prime }(\delta (F^{\prime }(y))))$$

where *δ* is a ReLU activation function, *F′* and *F*″ represent convolution operations with channel number 1 and convolution kernel size 1 × 1, respectively, which model inter-channel dependencies; and *σ* indicates the Sigmoid activation function that compresses the dynamic range of the input activation vector to [0, 1] to generate the calibrated vector *y′*, *y′_i_* indicates the importance of the *i*-th channel, The channel recalibration vector is used to recalibrate feature *X* to:

(4) $$M\;=\;[m_{1},m_{2},m_{3},\ldots ,m_{c}]\;=\;X\cdot y^{\prime }\;=\;[x_{1}{y^{\prime }}_{1},x_{2}{y^{\prime }}_{2},x_{3}{y^{\prime }}_{3},\ldots \;,{\;x}_{c}{y^{\prime }}_{c}]$$

Therefore, the weighting factor *M*, derived from the pure channel attention module, incorporates global contextual information and effectively enhances more discriminative features. The final output of the CAM is given as *M*.

#### 2.2.2. Multi-Scale Spatial Attention Module

**Figure 3:**
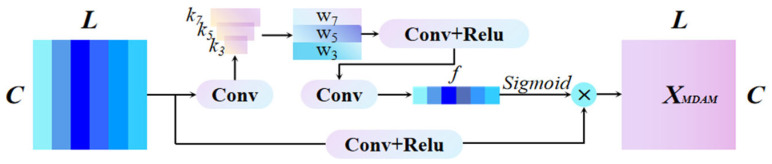
Spatial Attention Module.

Different types of seal end-face faults induce distinct AE signal characteristics. For example, localized cracks typically generate transient high-frequency pulses, while surface wear produces sustained low-frequency fluctuations. To effectively capture these diverse fault signatures in 1D AE signals, we introduce a spatial attention mechanism (SAM) that integrates multi-scale convolution with learnable weights. The proposed SAM employs three parallel convolutional kernels of different sizes to extract multi-scale features, thereby enhancing the detection of fault patterns across various temporal scales. The improved SAM architecture is shown in Figure 3.

The activation maps within convolutional layers encode the relative importance of different temporal signal segments. Consequently, the SAM employs a convolutional layer to perform a learnable weighting across all channel-wise activation maps. This process aggregates their features to precisely highlight the temporal segments most relevant to the fault. Specifically, assume that the input feature *T* is represented as $T\;=\;[t_{1},t_{2},t_{3},\ldots {,t}_{L}]$, where $t_{j}(t_{j}{\in \;R}^{1=L})$ corresponds to the *j*-th temporal signal location and j = 1, 2, . . . , L. First, the SAM obtains a temporal projection of feature T through a 1 × 1 convolutional layer with a single output channel, which is *s = W_agg_⋅T*, where *W_agg_* is a 1 × 1 convolution This operation performs a channel-wise aggregation of all activation maps in the input *T*. Then, features are extracted using three convolutional kernels with sizes k = [3, 5, 7], and the resulting multi-scale features are subsequently fused via a convolutional operation, formulated as:

(5) $$f^{\prime }=(W^{\prime \prime }\cdot \delta (W^{\prime }\cdot \left[\begin{array}{c} {\;f\;}_{3}=k_{3}\cdot s\\ f_{5\;}=k_{5}\cdot s\\ f_{7}=k_{7}\cdot s \end{array}\right]))$$

In this formulation, $W^{\prime }\in \;R^{3\times 3\times 1}$ represents the 1 × 1 convolutional with 3 input channels and 3 output channels, $W\prime \prime \in \;R^{1\times 3\times 1}$ represents a 1 × 1 convolution with 3 input channels and 1 output channels. The function *δ* represents the ReLU activation, and *σ* is the sigmoid activation, which compresses the dynamic range of the input activation vector to [0, 1]. The resulting attention weights form a temporal weight vector $f^{\prime }(f^{\prime }\in {\;R}^{1\times L})$, where each element *f′_j_* indicates the importance of the *j*-th time step. This vector *f′* is used to recalibrate the output features from the Channel Attention Module (CAM) via the following operation:

(6) $$N\;=\;[n^{1},n^{2},n^{3},\ldots {,n}^{L}]\;=\;M\cdot f^{\prime }$$

Before recalibration, a separable convolutional layer is first used to encode feature information between local time signal segments of *M*.

#### 2.2.3. Multi-Scale Dual Attention Module (MDAM)

The MDAM is a combination of the SAM and the CAM, with multi-scale primarily reflected in the design of the spatial attention module. It assigns different weights to both the channel features and time-series features of the input *X*. Additionally, a residual connection is introduced between the dual-attention modules to mitigate the degradation problem in deep networks. The fused output is given by:

(7) $$X_{\mathrm{MDAM}}\;=\;X\;+\;N$$

**Figure 4:**
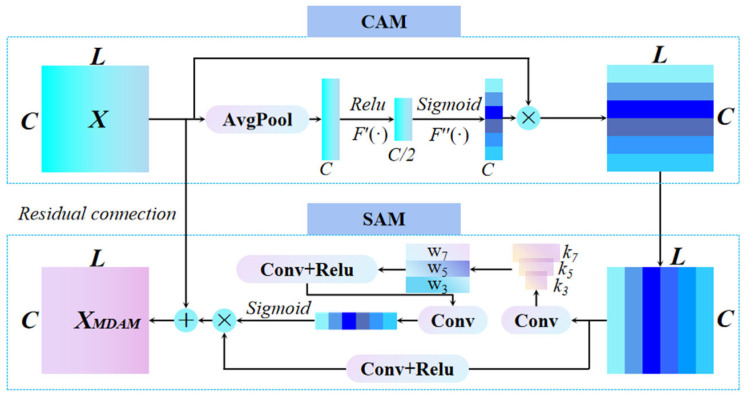
Multi-scale Dual Attention Module.

The basic structure of the MDAM is shown in Figure 4.

### 2.3. Fault Identification Method Based on MA1D-ResNet

This paper presents a fault diagnosis method using MA1D-ResNet, an architecture adapted from ResNet-18. To process the 1D time-series AE signals directly, we replace the 2D kernels with 1D convolutions and add a dropout layer for regularization. This allows the convolutional layers to autonomously learn features from the raw input.

**Figure 5:**
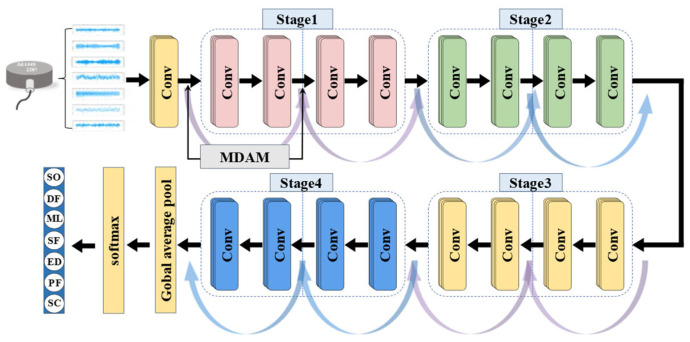
Network configuration of the MA1D-ResNet.

To explore the effect of attention module placement, we conducted ablation studies by inserting the Multi-scale Dual Attention Module (MDAM). The results indicate that the optimal configuration, which achieves the highest recognition accuracy, places the MDAM after both the initial convolutional layer and the first BasicBlock of Stage 1, forming a dual-path attention fusion, as shown in Figure 5. The network consists of subsequent stages, each containing two BasicBlocks, and ends with a global average pooling layer and a softmax output layer. The detailed configuration is provided in Table 1.

### 2.4. Construction of the Test Platform and Database

#### 2.4.1. Introduction to the Test Platform

**Figure 6:**
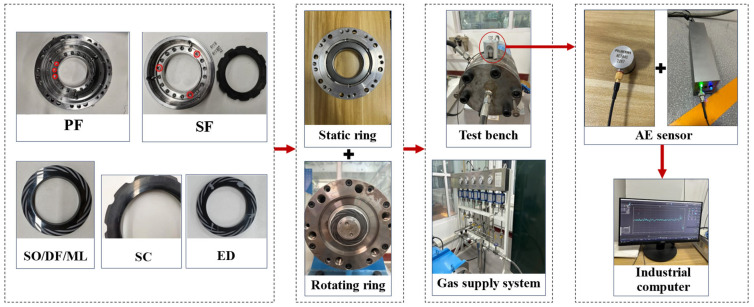
Dry gas sealed acoustic emission signal acquisition and testing system.

This study focuses on non-contact rotating seals and utilizes a custom-designed test platform capable of operating at speeds up to 3000 rpm, with a maximum design pressure of 8 MPa and compatibility for shaft diameters ranging from 60 to 180 mm. Figure 6 illustrates the acoustic emission (AE) signal acquisition system, which primarily consists of the main test rig, an AE acquisition unit, a gas circuit and supply system, and an industrial control computer.

The AE acquisition unit (Model: AE144S2287, Pengxiang Technology Co., Changsha, China) is mounted on the outer wall of the seal chamber, while the sensor (Model: PXDAQ24260B, Pengxiang Technology Co., Changsha, China) is equipped with a built-in low-noise preamplifier. All model training, testing, and validation were performed using a deep learning framework built upon PyTorch 2.6.0+cu118 and Python 3.9. The experiments were conducted on a workstation configured with a 13th Gen Intel^®^ Core™ i5-13600K processor and an NVIDIA GeForce RTX 2070 graphics card.

#### 2.4.2. Construction of Fault Database

The operating conditions for data acquisition of non-contact rotating seal status signals in this study are summarized in Table 2. All tests were conducted under a pressure of 0.1 MPa. During operation, the non-contact seal exhibits three distinct tribological states, a characteristic well-described by the Stribeck curve [39]. The optimal operating region of the system generally lies in the transition phase from mixed lubrication to hydrodynamic lubrication, where both friction and leakage are minimized [40]. Studies indicate that when the rotational speed reaches 1000 rpm, the tribological state of the non-contact seal approaches this transition point [41]. Therefore, with a normal seal ring, data were acquired using the AE sensor at motor speeds of 50 rpm and 600 rpm to represent start-stop fault signals. At 1000 rpm, AE signals were collected under both normal seal ring conditions and four types of end-face abnormal operation. A single-channel AE sensor was used with a sampling frequency of 1.25 MHz and a sampling duration of 6 s.

Deep learning models generally require substantial data for effective training. To expand the limited dataset and improve model generalization, this study employs a combination of data segmentation and Gaussian noise injection [42].

The original AE signal for each operating condition was acquired over a 6 s duration at a sampling frequency of 1.25 MHz, resulting in 7,500,000 data points per condition. This continuous signal was first segmented into 2000 sub-samples, each with a duration of 3 ms (3750 data points). These sub-samples were then partitioned into training, validation, and test sets in a ratio of 7:1:2. Consequently, for each fault category, this yielded 1400 training, 200 validation, and 400 test sub-samples.

**Figure 7:**
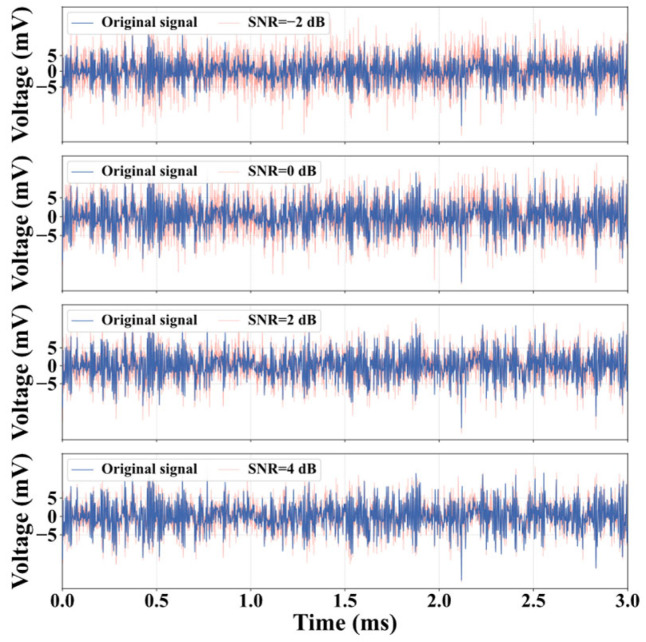
Gaussian denoising result.

To enhance the model’s generalization, data augmentation was applied only to the training set by injecting Gaussian white noise at four different Signal-to-Noise Ratio (SNR) levels: −2 dB, 0 dB, 2 dB, and 4 dB. This process expanded the number of training sub-samples for each category from 1400 to 7000 (1400 original × 5 variants [1 original + 4 noisy]). Ultimately, the final dataset comprised a total of 49,000 training samples (7 categories × 7000 samples), 1400 validation samples, and 2800 test samples. The experimental conditions for dry gas seal signal data acquisition under different states and the resulting training sample expansion are presented in Table 3. For illustration, the noise-injected versions generated from a normal operation signal are shown in Figure 7.

The noise addition method is described as follows:

(8) $$\mathrm{SNR}\;=\;10\log_{10}\frac{P_{\mathrm{signal}}}{P_{\mathrm{noise}}}$$

*P_signal_* and *P_noise_* denote the signal power and noise power, respectively. This approach effectively expands the original training set sample size by a factor of five. By introducing controlled noise into the training set, the training process significantly enhances the model’s robustness to noise interference.

### 2.5. Fault Diagnosis Process of Sealing End Face Based on MA1D-ResNet

**Figure 8:**
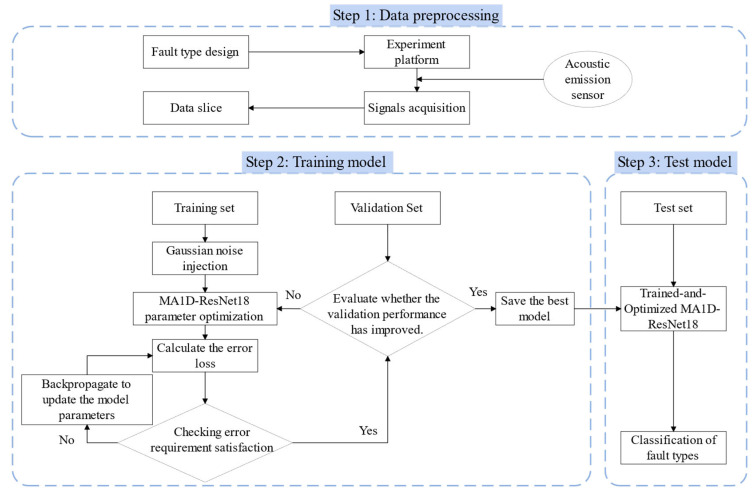
Identification process for seal face fault types.

This study introduces a novel hybrid framework that combines Multi-scale Attention models with Residual Neural Networks to address the challenges of limited datasets and noisy environments in fault diagnosis for Dry Gas Seal machines. The identification workflow is outlined in Figure 8.

(1) Signal Acquisition and Dataset Partition

The collected acoustic emission (AE) signals are divided into training, validation, and test sets in a 7:1:2 ratio. The training samples are then enhanced through Gaussian noise injection.

(2) Model Training

The MA1D-ResNet model is configured with the appropriate hyperparameters. During training, the Adam optimizer is used with the cross-entropy loss function. The initial learning rate is set to 0.001, with an adaptive decay strategy: if the loss does not decrease for five consecutive epochs, the learning rate is multiplied by 0.9. The total number of training epochs is set to 100. The specific hyperparameter settings for MA1D-ResNet are shown in Table 3. To further augment the dataset, gradient noise is injected into the training samples. The augmented training set is then fed into the model for forward propagation, where the prediction error is computed. If the error does not meet the predefined convergence criterion, backpropagation is triggered to update the network weights based on the calculated gradients. Once convergence is achieved, the model is evaluated on a separate validation set. If the accuracy meets the required threshold, the current network parameters and the best-performing model are saved. If the accuracy is unsatisfactory, the network parameters are adjusted, and the training process is restarted. All network weights are initialized using the Kaiming method [43].

(3) Fault Identification and Classification

The test set samples are fed into the optimized MA1D-ResNet model for fault identification, allowing the discrimination of various fault states.

## 3. Results

### 3.1. Visualization of Weights

**Figure 9:**
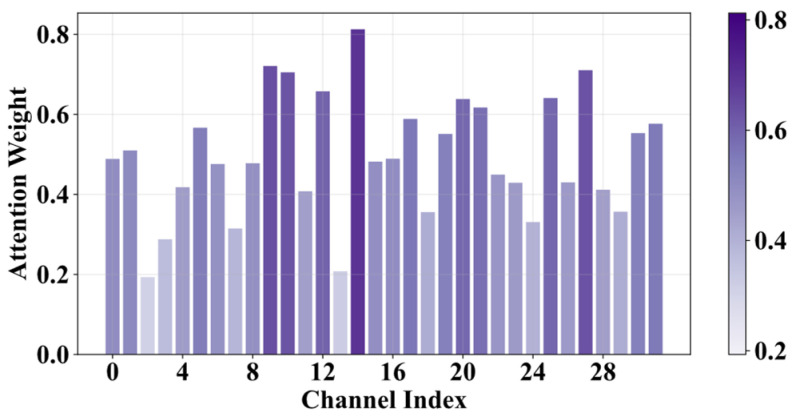
Visualization of Channel Attention Weights.

**Figure 10:**
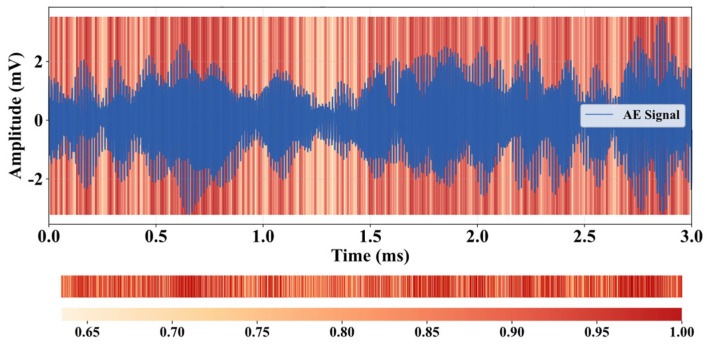
Visualization of Multi-scale Spatial Attention Weights.

To better understand the feature learning mechanism of the Multi-scale Dual Attention Module (MDAM), we use an input sample from a rotating ring end-face pit as an example. The multi-scale spatial attention module (SAM) and channel attention module (CAM) in Stage 1 of the MA1D-ResNet are visualized in Figure 9 and Figure 10. All spatial attention weights are extracted to match the length of the input signal. It can be observed that the SAM adaptively focuses on key signal segments, enabling the network to selectively learn the most important features, thereby improving both feature learning efficiency and capability. Meanwhile, the recalibration vectors from the CAM are visualized in Figure 10. The CAM encodes the relative importance of the 32 input channels, allowing the network to adaptively enhance the fault-related channels. The integration of SAM and CAM significantly boosts the network’s diagnostic performance, further demonstrating the necessity and effectiveness of both attention mechanisms in feature selection.

### 3.2. Performance Evaluation

**Figure 11:**
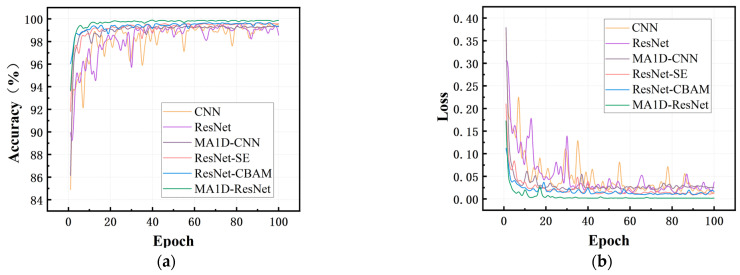
Accuracy rates and Loss of different models: (**a**) accuracy; (**b**) loss.

To demonstrate the superior accuracy of the proposed model, a comparative analysis was performed among six models: CNN [44], ResNet [45], MA1D-CNN, ResNet-SE [46], ResNet-CBAM [47], and MA1D-ResNet. In all cases, the attention modules were integrated at the same positions as in the proposed model. The accuracy and loss curves for these models on the validation set during training are shown in Figure 11.

All six models were trained for 100 epochs on a dataset of 49,000 samples using Python 3.9 and PyTorch 2.8.0+cu126. The MA1D-ResNet model completed training in approximately 1.21 h on an NVIDIA GeForce RTX 2070 GPU, with a total of 999,120 trainable parameters. It converged around the 20th epoch (0.23 h), demonstrating the computational efficiency of our approach in practical industrial applications.

As observed, the four models incorporating attention mechanisms exhibited better convergence and more stable training compared to the others. Among them, the proposed MA1D-ResNet displayed the most stable performance, with both accuracy and loss curves showing the fastest convergence and the best overall results.

Although all six models experienced some fluctuation during the initial training phase, their loss values on the validation set consistently showed a downward trend. After the 20th epoch, the proposed model stabilized, maintaining the lowest loss level. As training progressed, the accuracy of all models gradually improved and eventually converged, with the proposed model consistently achieving the highest accuracy, reaching 99.93% on the validation set. This comprehensive comparison underscores the clear superiority of the MA1D-ResNet approach in fault diagnosis for dry gas seal systems.

To ensure robustness, we conducted five repeated experiments. The MA1D-ResNet model was reinitialized with random seeds 42, 43, 44, 45, and 46 to update the initial weights. The model’s performance on the validation set is summarized in Table 4. We used three evaluation metrics: accuracy, precision, and recall, which are commonly used to assess classification performance. These metrics are defined as follows:

(9) $$\mathrm{Accuracy}\;=\;\frac{\mathrm{TP}\;+\;\mathrm{TN}}{\mathrm{TP}\;+\;\mathrm{FN}\;+\;\mathrm{FP}\;+\;\mathrm{TN}}\;\times \;100\%$$

(10) $$\mathrm{Precision}=\frac{\mathrm{TP}}{\mathrm{TP}+\mathrm{FP}}\;\times \;100\%$$

(11) $$\mathrm{Recall}=\frac{\mathrm{TP}}{\mathrm{TP}+\mathrm{FN}}\;\times \;100\%$$

Here, *TP*, *FP*, *TN*, and *FN* represent the number of true positives, false positives, true negatives, and false negatives, respectively. All three metrics range from 0 to 1, with higher values indicating better fault diagnosis performance.

In our results, the model’s Accuracy is equivalent to its macro-recall. This is because our test set was perfectly balanced, with 400 samples for each fault type, eliminating any class imbalance and ensuring a fair evaluation. Additionally, the close alignment of all performance metrics indicates that the model performs consistently across different measures, showcasing its excellence in this classification task.

**Figure 12:**
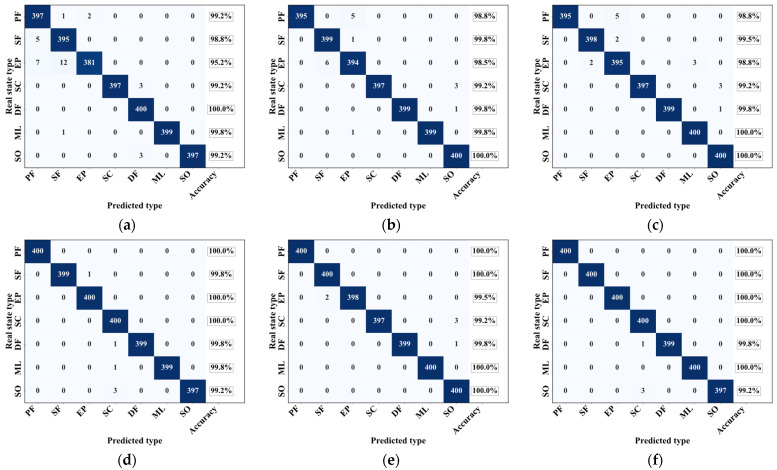
Confusion matrix heat maps and accuracy of different models: (**a**) CNN; (**b**) ResNet; (**c**) MA1D-CNN; (**d**) ResNet-SE; (**e**) ResNet-CBAM; (**f**) MA1D-ResNet.

To further evaluate the classification performance of the MA1D-ResNet model on the operational states of the non-contact seal, we used a confusion matrix on the test set. For comparison, we also calculated the classification accuracy, precision, and recall for the five baseline models: CNN, ResNet, MA1D-CNN, ResNet-SE, and ResNet-CBAM. The results are presented in Figure 12.

Among the six models evaluated, the proposed MA1D-ResNet achieved exceptional recognition accuracy for all seven operational states of the non-contact seal. It attained a perfect 100% recognition rate for five states (PF, SF, EP, ML, and SO), with only one DF sample being misclassified as SC. The SO state was the most challenging to identify across all models, as three SO samples were consistently misclassified as SC, indicating a significant similarity in the temporal characteristics between SO and SC. Despite this, the MA1D-ResNet achieved an average recognition accuracy of 99.86% on the test set, demonstrating superior diagnostic performance compared to all other models.

**Figure 13:**
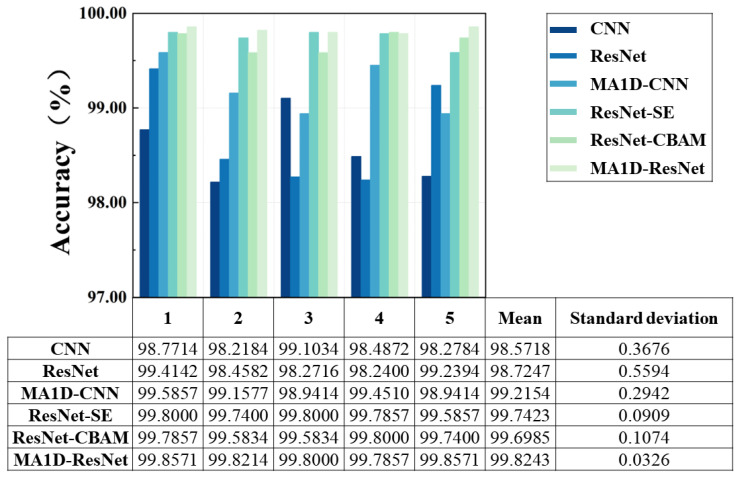
Classification accuracy and the standard deviation of five repeated experiments.

To ensure the reliability of the results, five repeated experiments were conducted for both the proposed MA1D-ResNet and the four baseline models, reducing the influence of randomness. As shown in Figure 13, MA1D-ResNet exhibited a marked improvement in accuracy compared to the model without an attention mechanism, indicating its higher sensitivity to initialization. In contrast, no significant differences were observed among the three attention-enhanced models. Among all the models, MA1D-ResNet achieved the highest mean accuracy and the smallest standard deviation. These results demonstrate that MA1D-ResNet outperforms the other five models, offering superior classification performance and enhanced robustness.

### 3.3. Analysis of Model Noise Resistance

In practical sealing systems, friction and collisions between rotating components generate high-intensity environmental noise, which reduces the signal-to-noise ratio (SNR) of the acquired acoustic emission (AE) signals and often masks critical fault features. To assess the model’s adaptability under realistic noisy conditions, noise was progressively added to the test set to simulate different noise environments. The recognition accuracy of each model was used as the key metric for evaluating noise robustness.

**Figure 14:**
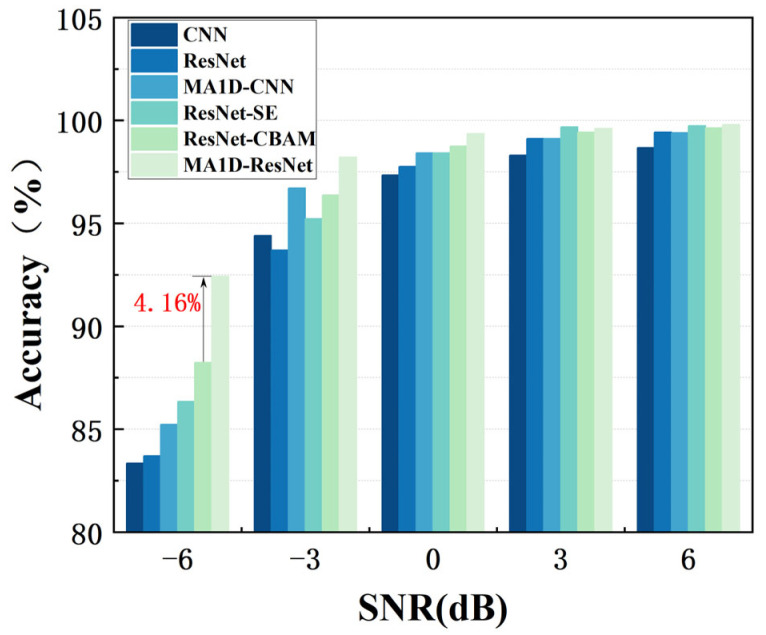
Comparison of anti-noise performance of different models.

To evaluate the model’s anti-noise performance, comparative experiments were conducted under sequentially set SNR levels of −6 dB, −3 dB, 0 dB, 3 dB, and 6 dB. Higher SNR values correspond to lower levels of noise interference in the signals. The results are presented in Figure 14 and Table 5, with the mathematical expression for calculating SNR provided in Equation (8).

The results demonstrate that the MA1D-ResNet model maintains a significant performance advantage under varying levels of noise interference. Even under challenging conditions with an SNR as low as −6 dB, the model achieves a recognition accuracy of 92.43%, outperforming the ResNet-CBAM model by 4.16 percentage points. Under moderate noise conditions (SNR of −3 dB), its accuracy improves to 98.21%. In environments with weaker noise (SNR levels of 0 dB, 3 dB, and 6 dB), the MA1D-ResNet model delivers even better performance, achieving accuracies of 99.36%, 99.61%, and 99.70%, respectively. These results clearly demonstrate that the proposed MA1D-ResNet network can effectively identify seal end-face fault types across a range of noise conditions, highlighting its remarkable generalization capability.

### 3.4. Impact of Data Augmentation Techniques on Model Noise Robustness

**Figure 15:**
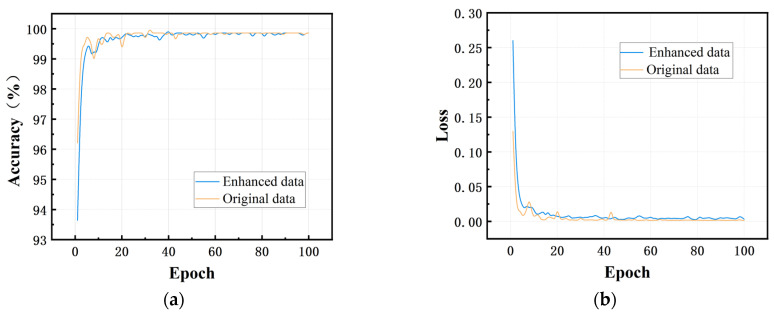
Comparison of training losses and accuracy: (**a**) accuracy; (**b**) loss.

The original dataset was augmented through segmentation and the injection of Gaussian noise. To evaluate the contribution of this augmentation to model robustness, we compared the proposed MA1D-ResNet against a baseline model trained solely on the unaltered dataset. As shown in Figure 15, the baseline model converged prematurely to a local optimum, although both models eventually achieved comparable accuracy and loss. This result confirms that incorporating Gaussian noise during training enhances the robustness of the learning process without compromising the model’s generalization capability.

**Figure 16:**
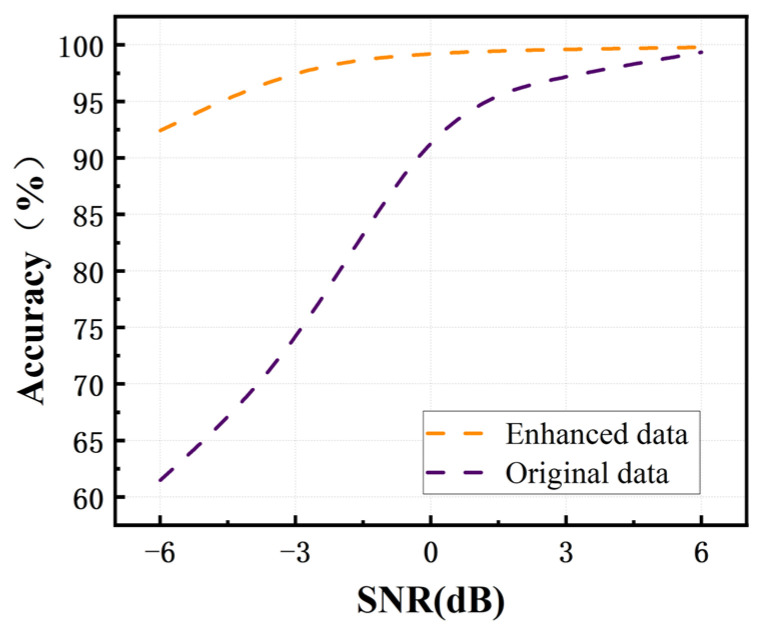
Accuracy comparison in a −6 dB environment.

For noise robustness evaluation, Gaussian noise was injected into the test set at signal-to-noise ratios (SNR) of −6 dB, −3 dB, 0 dB, 3 dB, and 6 dB. The recognition performance was then compared between models trained with and without Gaussian noise augmentation. The comparative accuracy results under different noise conditions are presented in Figure 16.

Adding Gaussian noise to data is not only a means of expanding the sample set, but the noise also serves as a regularization technique in data augmentation, forcing the model to learn more robust features rather than memorizing specific patterns of the training data. It is clearly seen that in a strong noise environment of −6 dB, the noise resistance performance of the training model with data augmentation is better.

### 3.5. t-SNE Visualization Analysis

**Figure 17:**
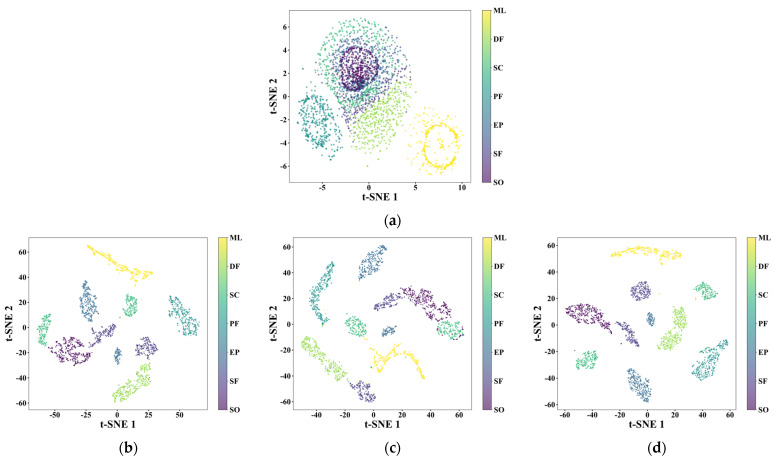

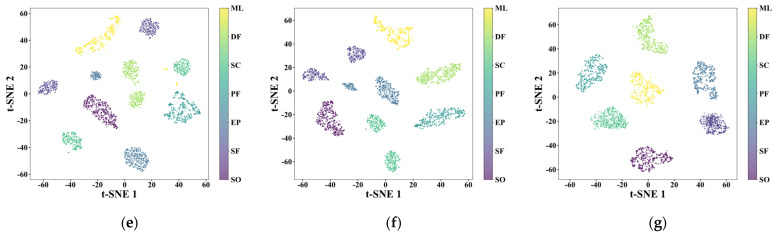
Visualizing 2D feature representations across MA1D-ResNet layers using t-SNE dimensionality reduction: (**a**) Input layer; (**b**) Conv1; (**c**) stage1; (**d**) stage2; (**e**) stage3; (**f**) stage4; (**g**) Output layer.

To gain deeper insights into the feature extraction mechanism of the MA1D-ResNet model at various hierarchical levels, we employed the t-SNE algorithm to visualize feature representations from the input layer and each subsequent stage layer. The visualizations, shown in Figure 17, clearly illustrate the model’s evolving discriminative capability for fault features as the network deepens. Different fault categories are represented by distinct colors.

Initially, the raw input features exhibit substantial overlap with blurred class boundaries, making effective classification difficult due to the inherent complexity and noise interference in acoustic emission signals. After processing by the initial convolutional layer, which extracts fundamental temporal patterns through localized filtering, features from different categories begin to separate and gradually form clusters, indicating the model’s capacity for basic feature discrimination. The integration of Stage 1 and the Multi-scale Dual Attention Module (MDAM) further enhances feature discriminability by adaptively weighting both channel-wise and spatial-temporal characteristics, allowing for preliminary differentiation among the seven fault patterns through multi-scale feature fusion. As the network depth increases, the hierarchical architecture progressively refines feature representations through residual learning, with the clustering effect continuously improving as more discriminative features are extracted at different abstraction levels. By the final classification layer, samples from the same category form compact clusters in the feature space, with clear and well-separated boundaries between different fault states, demonstrating that the model has effectively learned noise-invariant and discriminative representations of various fault states, achieving optimal classification performance.

Thus, the MA1D-ResNet-based deep learning model, through its hierarchical feature learning and adaptive attention mechanisms, enables efficient and accurate identification of operational states in non-contact seals even under challenging noise conditions.

## 4. Conclusions and Prospects

The core industrial value of this study lies in providing a highly robust and practical solution for early fault diagnosis of dry gas seals in high-noise environments. This method facilitates the transition from traditional “scheduled maintenance” or “reactive repair” to more advanced “predictive maintenance” in industrial settings. By enabling early fault detection, it significantly reduces unplanned downtime, prevents safety incidents such as media leakage caused by seal failure, and optimizes maintenance cycles and spare parts inventory. This approach offers crucial technical support for ensuring the long-term, safe, and stable operation of critical machinery. The main conclusions are as follows:

(1) The proposed model demonstrates excellent performance in dry gas seal fault classification, achieving an accuracy of 99.8571% on the test set. Under identical parameter settings, it significantly outperforms other 1D-CNN-based models, including CNN, ResNet, MA1D-CNN, ResNet-SE, and ResNet-CBAM, exhibiting both higher accuracy and superior convergence.
(2) Comparative experiments with six different deep learning models under noisy conditions confirm that the proposed model maintains high recognition accuracy and strong noise resistance, even under substantial noise interference.
(3) The use of data augmentation techniques—specifically data segmentation and Gaussian noise injection—substantially enhances the model’s generalization ability and noise robustness. Comparative experiments confirm that the augmented model outperforms the non-augmented version in terms of accuracy, training loss, and anti-noise performance.

This study relies solely on acoustic emission (AE) signals, which limits data diversity and constrains further improvement of model robustness under strong noise. Additionally, the current fault database covers only four structural fault types and should be expanded to include other modes such as seal ring deformation, impurities in the gas film, and insufficient or failed lubrication. Future work will focus on: developing a multi-source signal fusion diagnosis framework integrating AE and vibration signals; designing a deep learning model based on multi-head attention to significantly enhance noise robustness; and expanding the fault database to include more failure modes. Furthermore, exploring hybrid frameworks that integrate the proposed data-driven model with model-based approaches, such as discrete-event systems, presents a promising research direction.

## Figures and Tables

**Table 1:** Network configuration of the MA1D-ResNet architecture.

Layer	Type	Kernel	Channel	Stride	Attention	Output
0	Input					3750 × 1
1	Conv	7 × 1	16	2	MDAM	3750 × 16
2	Stage1	3 × 1	32	2	MDAM	1875 × 32
3	Stage2	3 × 1	64	2		938 × 64
4	Stage3	3 × 1	128	2		469 × 128
5	Stage4	3 × 1	256	2		235 × 256
Global average pooling and softmax						
SO DF ML SF ED PF SC						

Footnote: SO—Stable operation; DF—Dry friction; ML—Mixed lubrication; SF—Spring failure (uniform distribution); ED—Rotary ring end face dent; PF—Partial spring failure; SC—Scratch on end face of static ring.

**Table 2:** Dry gas seal signal data acquisition test under different conditions and training sample augmentation results.

Operating Condition	Category	Abbreviation	Training Sample	Speed (rpm)	SNR (dB)	Lable Index
Normal	Stable operation	SO	7000	1000	−2, 0, 2, 4	0
Structural failure	Spring failure (uniform distribution)	SF	7000	1000	−2, 0, 2, 4	1
	Partial spring failure	PF	7000	1000	−2, 0, 2, 4	2
	Rotary ring end face dent	ED	7000	1000	−2, 0, 2, 4	3
	Scratch on end face of static ring	SC	7000	1000	−2, 0, 2, 4	4
Start-stop failure	Dry friction	DF	7000	50	−2, 0, 2, 4	5
	Mixed lubrication	ML	7000	600	−2, 0, 2, 4	6

**Table 3:** Hyperparameter optimization settings for MA1D-ResNet.

Hyperparameter	Search Range	Optimal Value	Remarks
Learning Rate	[1 × 10^−4^, 5 × 10^−4^, 1 × 10^−3^, 5 × 10^−3^]	1 × 10^−3^	Adam optimizer
Batch Size	[32, 64, 128]	64	Balance of convergence a
Channel Reduction Ratio	[2, 4, 8]	2	Channel attention compression
Dropout Rate	[0.2, 0.3, 0.5]	0.5	Final classification layer
Attention Dropout	[0.1, 0.2, 0.3]	0.2	Attention fusion module
Kernel Sizes	[3, 5, 7], [5, 7, 9], [7, 9, 11]	[3, 5, 7]	Multi-scale spatial attention

**Table 4:** Five repeated experiments.

Indicators	Seed (42)	Seed (43)	Seed (44)	Seed (45)	Seed (46)	Mean	Standard Deviation
Accuracy	99.7500%	99.7857%	99.8214%	99.7857%	99.8571%	99.8000%	0.0364%
Precision	99.7522%	99.7876%	99.8229%	99.7873%	99.8586%	99.8017%	0.0362%
Recall	99.7500%	99.7857%	99.8214%	99.7857%	99.8571%	99.8000%	0.0364%

**Table 5:** Comparison of anti-noise performance of different models.

SNR (dB)	CNN (Acc%)	ResNet (Acc%)	MA1D-CNN (Acc%)	ResNet-SE (Acc%)	ResNet-CBAM (Acc%)	MA1D-ResNet (Acc%)
−6	83.35	83.70	85.23	86.35	88.25	92.41
−3	94.41	93.70	96.71	95.23	96.37	98.21
0	97.353	97.75	98.42	98.42	98.75	99.36
3	98.31	99.12	99.12	99.68	99.42	99.61
6	98.68	99.42	99.39	99.74	99.64	99.79

## Data Availability

The raw data supporting the conclusions of this article will be made available by the authors on request.
